# Therapeutic Efficacy of 2% Mupirocin in Managing Staphylococcus aureus and Streptococcus pyogenes Wound Infections

**DOI:** 10.7759/cureus.82366

**Published:** 2025-04-16

**Authors:** Paola Rivera, Marc B Bello, Geraldo Brito, Gabriela Pichardo, Elias Cruz, Daisy Blanco

**Affiliations:** 1 Dermatology, Instituto Dermatológico y Cirugía de Piel “Dr. Huberto Bogaert Díaz”, Santo Domingo, DOM

**Keywords:** antibiotic resistance, infected wounds, mupirocin, staphylococcus aureus, streptococcus pyogenes, topical therapy

## Abstract

Background

Skin and soft tissue infections are a significant healthcare concern, ranging from mild cases treated with topical therapy to severe infections requiring systemic antibiotics and surgical intervention. The increasing global antibiotic resistance, particularly involving *Staphylococcus aureus* and *Streptococcus pyogenes*, complicates the management of these infections. Mupirocin, a topical antibiotic, has shown remarkable efficacy in treating infections caused by these pathogens. This quasi-experimental study aimed to evaluate the short-term efficacy and safety of 2% topical mupirocin cream in treating superficial traumatic and surgical wounds infected with *S. aureus* and/or *S. pyogenes* among patients, predominantly pediatric, at the Dominican Dermatological Institute and Skin Surgery “Dr. Huberto Bogaert Díaz.” Efficacy was assessed using the Severity of Infected Wounds Scale (SIRS), and safety was determined based on adverse effects over an 11-day follow-up period. The study seeks to inform real-world management strategies and reduce reliance on systemic antibiotics.

Methodology

A quasi-experimental study was conducted between January and April 2023 at the Dominican Dermatological Institute and Skin Surgery “Dr. Huberto Bogaert Díaz.” Patients presenting with clinically infected wounds were enrolled to assess the efficacy of 2% mupirocin cream. The intervention involved applying the cream three times daily for 10 days. The primary outcomes were therapeutic response and incidence of adverse effects. Therapeutic response was assessed using SIRS, which evaluates erythema, swelling, discharge, and local warmth, each scored from 0 (absent) to 3 (severe), for a total score ranging from 0 to 12. Baseline scores were recorded on day one, and follow-up scores on day 11. A therapeutic response was defined as a reduction of 50% or more in the total SIRS score compared to baseline. Adverse effects were monitored through physical examination and patient interviews.

Results

Of the 135 patients treated, 134 (99.2%) exhibited a favorable therapeutic response without adverse effects. Only one (0.7%) patient developed an allergic reaction, characterized by a skin rash, which led to the discontinuation of the treatment. *S. aureus* was isolated in 76 (56.3%) patients, *S. pyogenes* in 3 (2.2%) patients, and both pathogens in 56 (41.5%) patients.

Conclusions

The study confirms that 2% mupirocin cream is highly effective and safe for treating superficial wounds infected by *S. aureus* and/or *S. pyogenes*. The low incidence of adverse reactions suggests that mupirocin can be considered a first-line topical therapy for such infections, potentially reducing the need for systemic antibiotics and mitigating the risk of systemic antibiotic resistance.

## Introduction

Skin and soft tissue infections account for a significant percentage of healthcare consultations. These infections can range from mild cases, managed with topical treatment, to those with severe systemic involvement, requiring systemic antibiotic therapy and even surgical debridement. Generally, skin and soft tissue infections result from an imbalance between the defense mechanisms of the skin barrier and the virulence and pathogenicity factors of the microorganisms affecting it [1].

The global increase in antibiotic resistance has been exacerbated by the misuse and overuse of available antimicrobials. This phenomenon is particularly concerning in the context of skin and soft tissue infections, where the most prevalent bacteria are *Staphylococcus aureus* and *Streptococcus pyogenes*, although Gram-negative bacilli are also observed, especially in hospitalized patients [1]. The most predominant bacteria are *S. aureus*, *S. pyogenes*, and Gram-negative bacilli in hospitalized patients.

*S. aureus* is a Gram-positive coccus that clusters in grape-like formations and is the primary pathogen involved in skin and soft tissue infections. Its ability to evade the immune system, by secreting molecules that inhibit neutrophil migration, block phagocytosis, or alter apoptotic pathways, allows it to generate a wide range of clinical manifestations, from folliculitis to life-threatening necrotizing infections. The prevalence of methicillin-resistant *Staphylococcus aureus* (MRSA) varies significantly in the South American region, with reported rates as high as 88% in Argentina and much lower in Brazil (4.5-8.6%) and Bolivia (1.5%) [1].

On the other hand, *S. pyogenes* is the second most prevalent bacterium found in cutaneous and subcutaneous infections. This Gram-positive coccus, which forms chains, is present in both mucous membranes and on skin and can cause a wide variety of manifestations, from superficial cutaneous infections such as erysipelas to systemic manifestations such as severe bacterial pneumonia and meningitis. Its virulence is enhanced by the production of pathogenic factors such as streptolysin S and O, hyaluronidase, streptokinase, and pyrogenic exotoxins [1].

Wounds represent a disruption of the skin’s structural, morphological, and anatomical integrity, which can be caused by trauma and surgical interventions, among other factors. Depending on their severity, wounds can present varying degrees of complications. Healing is the physiological process by which the loss of skin continuity caused by a wound is resolved. This complex process involves several cell types that act sequentially and overlap, attracted by cytokines, and is influenced by both general factors, such as blood circulation, nutrition, or pre-existing conditions, and local factors, such as contamination of the affected area, temperature, or dehydration [2].

The healing process consists of three differentiated phases, namely, the inflammatory phase, the proliferative phase, and the remodeling phase. These phases aim to remove pathogens and damaged tissue and regenerate the tissue to gradually reduce the wound. During the remodeling phase, a scab forms because collagen production exceeds its degradation. This process is marked by the action of matrix metalloproteinases, specifically collagenases, whose function is to degrade collagen. However, this process can be hindered by factors such as wound desiccation, bacterial contamination, necrosis, pressure, trauma, or edema, which can result in contractured/normotrophic, atrophic, hypertrophic, or keloid scars. Various groups of active principles, including antibiotics and re-epithelialization agents, are used to promote and facilitate the healing process [2].

It is expected that all wounds are colonized by microbes; however, this does not imply or indicate the presence of an acute infectious process. Therefore, antibiotic therapy is not indicated for all wounds and should be reserved only for those showing clinical signs of infection. There is no published evidence supporting antibiotic therapy as “prophylaxis” in non-infected chronic wounds, nor for improving the healing potential of wounds without clinical evidence of infection. Clinical signs of infection in a wound that could justify antibiotic therapy include local signs, such as cellulitis, lymphangitic streaking, purulent discharge, foul odor, wet gangrene, and osteomyelitis, and systemic symptoms, such as fever, chills, nausea, hypotension, hyperglycemia, leukocytosis, or changes in the mental status [3].

Mupirocin, also known as pseudomonic acid A, is a secondary metabolite produced by the Gram-negative bacterium *Pseudomonas fluorescens*. This topical antibiotic works by binding to bacterial isoleucyl-tRNA synthetase, inhibiting protein synthesis in microorganisms. Its primary use is in treating infections caused by pathogens such as strains of *Streptococcus* and *Staphylococcus*, including those resistant to methicillin. Specifically, mupirocin is widely used in treating MRSA, a pathogen of relevance in wound infections and one of the leading causes of nosocomial bloodstream infections. Additionally, mupirocin has minimal impact on the normal skin flora, including *Propionibacterium*, which could help preserve the skin’s natural defense mechanisms [4].

Mupirocin exhibits bacteriostatic properties at low concentrations but possesses bactericidal activity after 24 hours of topical application (20,000 μg/mL with a 2% formulation). Moreover, its antibacterial property is pH-dependent, meaning that at an acidic pH, it shows amplified in vitro antibacterial activity, making the skin’s low pH a favorable environment for effective treatment [5].

Although studies on wound healing have primarily focused on mupirocin’s antibacterial potential, some studies have reported its effects on inflammation and cell migration. Twilley et al. (2022) demonstrated that mupirocin stimulated the production of tumor necrosis factor (TNF)-α in RAW 264.7 cells [4]. TNF-α is a critical cytokine involved in the inflammatory stage of wound healing. Furthermore, the wound healing process in mice was delayed when treated with anti-TNF-α monoclonal antibodies, resulting in decreased inflammatory cell and fibroblast density. Conversely, TNF-α administration significantly improved wound closure. This suggests mupirocin’s potential in wound healing through a mechanism distinct from its antibacterial activity [4].

In the Dominican Republic, updated information on the incidence, prevalence, and statistics of surgical wounds is limited. A study conducted in 1992 at the Robert Reid Cabral Hospital revealed that out of 541 surgically operated patients, 6% (32 cases) had infected surgical wounds. These data, while valuable, reflect a situation that has since changed and highlight the lack of updated information in the national context. The high proportion of infected wounds reported in the study underscores the need for recent research. An updated study could provide more accurate data on the incidence of infected wounds, both of surgical and non-surgical etiology, as well as on the most common etiological agents and their behavior. The information obtained would significantly contribute to a better understanding and management of wound infections in the country.

Wound infections represent a considerable challenge for the healthcare system due to their economic and public health implications. Early detection and effective treatment are essential to reduce the impact of these infections, especially in a scenario where antibiotic resistance is on the rise.

Topical antibiotics, such as mupirocin, play a crucial role in managing localized skin infections. These topical treatments provide an effective alternative to systemic antibiotics and are a key tool in dermatology. However, prolonged use of these antibiotics can lead to bacterial resistance, highlighting the importance of their controlled and well-regulated application, supported by therapeutic guidelines.

In this context, the following clinical question arises: how does 2% mupirocin perform in the efficacy of treating wounds infected by *S. aureus* and/or *S. pyogenes* in patients at the Instituto Dermatológico Dominicano y Cirugía de Piel “Dr. Huberto Bogaert Díaz” from January to April 2023. This study seeks to evaluate the efficacy of local treatment in a specific setting and provide relevant data to improve the management of skin infections.

## Materials and methods

Study type and location

This quasi-experimental study was conducted without a control group and included patients with wounds infected by *S. aureus* and/or *S. pyogenes*. This study, conducted between January and April 2023 at the Dominican Dermatological Institute and Skin Surgery Center “Dr. Huberto Bogaert Díaz,” aimed to assess the therapeutic effects of mupirocin.

Study participants

The study population consisted of patients over 18 months of age with sutured or lacerated wounds showing clinical evidence of infection, who attended the dermatology consultation clinic at the Dominican Dermatological Institute and Skin Surgery “Dr. Huberto Bogaert Díaz” between January and April 2023. The patients were prospectively enrolled and treated during the study period. Institutional Review Board approval was obtained before study initiation, and written informed consent was secured from all participants or their legal guardians.

Inclusion criteria

The data included complete records of patients aged 18 months or older with superficial wounds no longer than 10 cm in length and no more than 100 cm³ in area, who exhibited signs of infection, had a score of 8 or higher on the Skin Infection Severity Scale (see Appendices), had not received prior treatment, and provided signed informed consent. Additionally, these patients attended the dermatology consultation during the aforementioned period.

Exclusion criteria

Patients with the following conditions were excluded from the study: pregnant women, breastfeeding mothers, infected wounds caused by bites, patients who had undergone any prior surgical treatment for the infection, such as drainage or debridement, or who had used any medication beforehand. Additionally, those with systemic or dermatological conditions that could interfere with the healing process, such as diabetes mellitus, liver diseases, arterial hypertension, heart failure, kidney failure, or Ehlers-Danlos syndrome, were excluded, as well as those who did not exhibit systemic symptoms of infection before or during the study.

Retention criteria

Patients included in the study had bacteriological cultures confirming the isolation of *S. aureus*, *S. pyogenes*, or both, without presenting signs or symptoms of systemic infection. Eligible patients were recruited and followed a standard therapeutic regimen according to Mayo Clinic guidelines for 2% mupirocin ointment. Their outcomes were evaluated and included as part of the post-therapeutic assessment results.

Data collection instrument

The data collection instrument used (available in Appendices) was a form created by the researchers with the variables to be measured. It was filled out based on the information obtained from the patients during the medical interview conducted by the researchers. The data collection technique was direct. The patient was first questioned, verifying the presence of the wound and its characteristics, to determine if they met the inclusion criteria for the study. Subsequently, the patient was asked if they wished to voluntarily participate in the study. Once their response was positive, they signed an informed consent document. After the document was signed, the information was gathered using a questionnaire that inquired about the general patient data and the clinical characteristics of the wound. Likewise, the wound’s score was determined using the Infected Wound Severity Scale (see Appendices), and, subsequently, the wound culture was obtained. All information was recorded in pre-assessment and therapeutic follow-up forms. Subsequently, a follow-up visit was conducted five days after the culture was obtained to evaluate its results and assess whether the patient would continue in the study or not. Then, a third visit was scheduled on day 11 to evaluate whether the treatment was effective or not.

Procedure

After obtaining informed consent, photographs of each lesion were taken. Patients were provided with a neutral soap (without antiseptic properties) and 2% mupirocin topical cream, along with detailed instructions on their use. They were instructed to gently cleanse the wound with neutral soap, pat it dry, and apply a thin layer of mupirocin 2% cream until fully absorbed, three times a day for 10 days. A wound sample was collected on the first day of treatment. Five days later, patients returned for a control visit to review culture results and confirm eligibility to continue. On day 11, the effectiveness of the treatment was evaluated.

Data tabulation

The collected data were meticulously reviewed and organized for manual processing. These data were then subjected to statistical analysis, calculating simple and relative frequencies, as well as means and percentages.

Analysis

The analysis was conducted considering the parameters established in the Severity of Infected Wounds Scale (SIRS) to evaluate whether clinical healing was achieved. The areas where the wounds were located were photographed using a digital camera to assess the wound’s progression before, during, and after the treatment.

## Results

Of the 135 wounds evaluated, the vast majority (93.3%) were of traumatic origin, while only 6.7% were surgical. Within traumatic injuries, wounds caused by sharp objects represented the highest proportion (31.9%), closely followed by blunt object trauma (28.9%) and post-fall injuries (31.1%). Laceration wounds were the least frequent type of trauma-related injury (1.5%). Surgical wounds consisted entirely of sutured incisions (Table 1).

**Table 1:** Classification of wound types by etiology.

Etiology	Type of wound	Cases (%)
Traumatic injury	Post-fall injury	42 (31.1)
	Sharp object wound	43 (31.9)
	Laceration wound	2 (1.5)
	Blunt object wound	39 (28.9)
Surgical	Sutured wound	9 (6.7)
Total		135 (100.0)

The study included 135 patients, with 78 females (57.8%) and 57 males (42.2%). A large majority of participants (77%) were under the age of 20 years. The most represented age group was 1-5 years, accounting for 56.4% of females and 66.7% of males (Table 2). Representation decreased with increasing age, with only a small proportion of participants over 30 years old. These data reflect the high burden of wound infections in the pediatric population, particularly among preschool-aged children.

**Table 2:** Characteristics of the study population.

Age (years)	Sex			
	Female (n = 78)	%	Male (n = 57)	%
1–5	44	56.4	38	66.7
6–10	16	20.5	7	12.3
11–20	10	12.8	6	10.5
21–30	5	6.4	5	8.8
31–40	1	1.3	1	1.8
41–50	2	2.6	0	0

**Figure 1:**
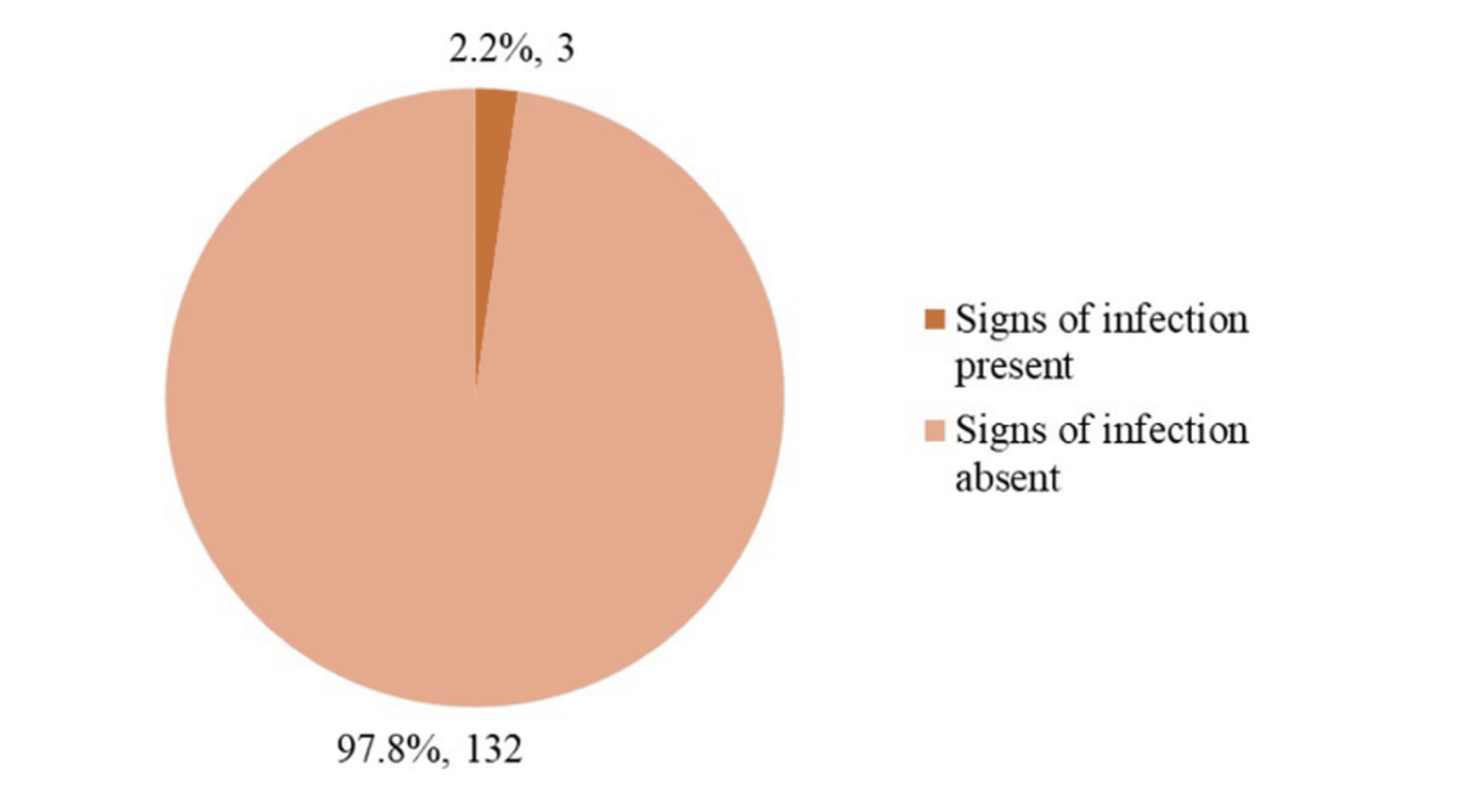
Therapeutic response to 2% mupirocin in treating infected wounds (n = 135).

Figure 1 illustrates the therapeutic response to 2% mupirocin application. Overall, 97.8% of patients achieved a favorable outcome, indicated by resolution of infection, while 2.2% continued to exhibit clinical signs of infection.

**Figure 2:**
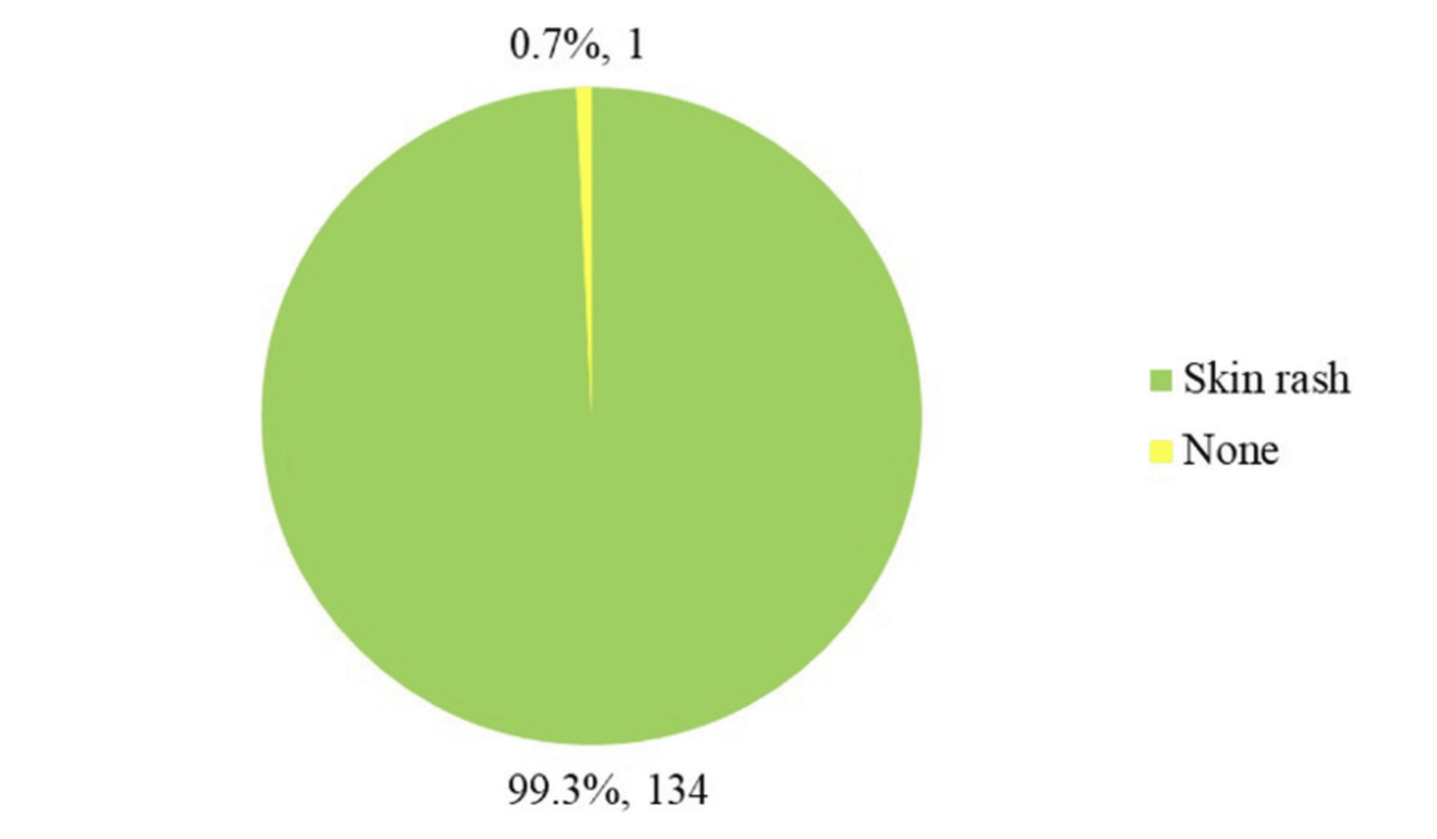
Incidence of a rash as an adverse effect following 2% mupirocin application (n = 135).

As shown in Figure 2, adverse effects were minimal, with only 0.7% developing a skin rash, whereas 99.3% experienced no adverse reactions.

**Figure 3:**
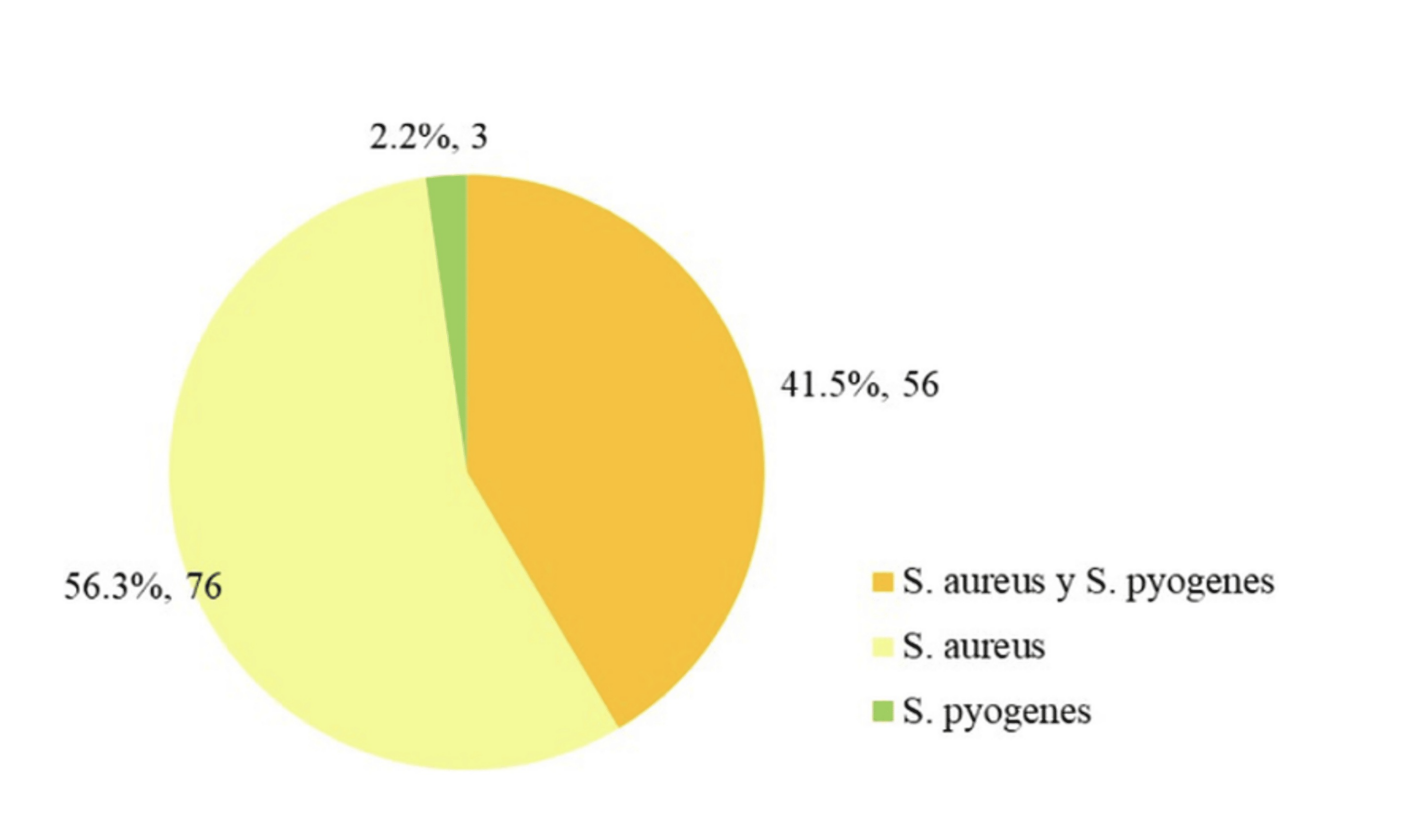
Distribution of isolated microorganisms in infected wounds (n = 135).

Figure 3 depicts the microorganisms isolated, with 56.3% yielding *S. aureus*, 41.5% both *S. aureus* and *S. pyogenes*, and 2.2% *S. pyogenes* alone.

**Figure 4:**
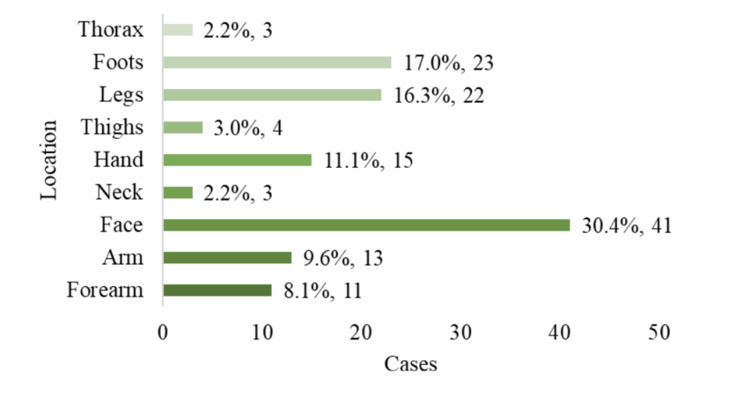
Anatomic distribution of wound locations (n = 135).

Figure 4 illustrates the wound locations. The face was the most commonly affected site (29.3%), followed by the feet (17.0%) and legs (16.3%). The hands, arms, and forearms accounted for 11.1%, 9.6%, and 8.1% of cases, respectively; the thigh comprised 3.0%, and the neck and chest each 2.2%.

## Discussion

Recent studies have highlighted the substantial efficacy of 2% mupirocin in treating infected wounds, particularly those complicated by *S. aureus*. In a pivotal 2021 study, patients with dermatoses secondarily infected by *S. aureus* were treated with topical 2% mupirocin, which was shown to be highly effective and safe in eradicating the bacterial infection. The study’s findings strongly suggested that mupirocin can effectively resolve skin infections, positioning it as a valuable alternative to systemic antibiotics in managing localized infections [5]. This is particularly relevant in an era where antibiotic resistance poses a significant threat to global health, underscoring the need for effective topical treatments.

The growing issue of systemic antibiotic overuse, particularly in wounds without definitive signs of infection, cannot be overlooked. Inappropriate use of systemic antibiotics not only contributes to the rise of antibiotic-resistant strains but also exposes patients to unnecessary risks associated with systemic drug therapy. This situation accentuates the importance of considering topical antibiotics such as mupirocin as the first-line treatment for localized skin infections. Comparative studies between topical mupirocin and systemic antibiotics, such as cephalexin, have demonstrated that mupirocin is equally effective in resolving bacterial infections, with the added benefit of a more favorable side effect profile. Moreover, mupirocin’s topical application minimizes the risk of systemic adverse effects commonly associated with oral or intravenous antibiotic therapies, which further enhances its appeal in clinical practice [6].

The cost-effectiveness of mupirocin is another factor that makes it an attractive option for treating skin infections. The financial burden of healthcare is a significant concern, particularly in resource-limited settings. The ability to achieve similar, if not superior, clinical outcomes with mupirocin compared to systemic antibiotics, while also reducing treatment costs, provides a compelling argument for its broader use in clinical settings. This cost saving is particularly relevant for healthcare systems globally, as they strive to manage limited resources effectively while maintaining high standards of care [1,2].

Adverse reactions associated with mupirocin remain minimal, with fewer than 1.5% of patients reporting mild symptoms such as a burning sensation or localized pain at the application site. Other adverse reactions, such as itching or skin rashes, are exceedingly rare, further reinforcing the safety and tolerability of mupirocin in routine clinical practice. The low incidence of side effects associated with mupirocin not only enhances patient compliance but also supports its use as a safer alternative to systemic antibiotics, which can carry a higher risk of adverse effects [7].

When comparing these findings with results from previous studies, the efficacy and safety of 2% mupirocin in treating skin infections caused by *S. aureus* and *S. pyogenes* are further substantiated. These results build a strong case for prioritizing the use of topical antibiotics such as mupirocin, particularly in settings where systemic infections are not evident. Systemic antibiotics should be reserved for cases with clear signs of systemic infection, such as fever, leukocytosis, or bacteremia. In contrast, mupirocin can be effectively used both prophylactically and therapeutically in managing infected wounds or those at high risk of infection, thus optimizing infection control strategies [3,4,8]. This approach not only maximizes the therapeutic benefits for patients but also contributes to a broader effort to combat the global challenge of antibiotic resistance by reducing the unnecessary use of systemic antibiotics. Such practices promote a more judicious and sustainable approach to infection management in both inpatient and outpatient settings.

This study has important limitations that must be considered when interpreting the findings. First, the lack of randomization and blinding introduces the possibility of selection and observer biases, which may have influenced both the recruitment of participants and the assessment of outcomes. These methodological limitations were primarily due to logistical and resource constraints. However, to help mitigate observer bias, we employed SIRS, a standardized and structured clinical tool used consistently across all patients to assess infection severity and therapeutic response objectively.

Additionally, the study was conducted at a single dermatological center in the Dominican Republic, which limits the generalizability of the results to other clinical settings or populations with different demographic or epidemiological profiles. This limitation is further compounded by the high proportion of pediatric patients, particularly those under 20 years of age, which narrows the applicability of the findings to older age groups.

Another limitation is the relatively short follow-up duration of 11 days, which may be insufficient to assess long-term outcomes such as relapse or reinfection. This period was selected to capture immediate post-treatment clinical response in accordance with typical topical therapy protocols, but future studies should consider extended follow-up to evaluate sustained resolution and recurrence risk.

Resistance profiling was not performed on the bacterial isolates. Specifically, no screening for MRSA or mupirocin resistance was conducted. This was due to laboratory and resource limitations during the study period. As such, the efficacy of mupirocin in the context of antimicrobial resistance could not be fully assessed. Future investigations should incorporate susceptibility testing to better understand mupirocin’s clinical utility in resistant infections.

Further, because of the quasi-experimental design without a control group and the absence of inferential statistical analyses, the conclusions drawn from this study should be interpreted with caution. While the high therapeutic response rate and low incidence of adverse effects are promising, these findings must be considered exploratory rather than definitive. Without a comparator group, it is not possible to fully exclude the influence of natural healing or other confounding factors. A more rigorous design, such as randomized controlled trials involving diverse populations, broader microbiological evaluation, and longer follow-up periods would strengthen the evidence base. Acknowledging these methodological constraints more critically enhances the transparency and reliability of the study’s conclusions.

## Conclusions

Superficial wounds resulting from trauma or surgical procedures that later develop into infections, primarily caused by *S. aureus* or *S. pyogenes*, respond well to treatment when using 2% mupirocin ointment exclusively. The occurrence of skin rashes was observed in just one out of the 135 patients examined, indicating that adverse reactions to the medication are rare and seldom lead to stopping treatment. These superficial wounds were found in various areas of the body, excluding the glutes and external genitalia, with the majority of cases affecting the face, feet, and legs. Overall, the ages of the patients ranged from 18 months to 50 years, with most cases being in children. Specifically, 105 out of 135 cases involved patients under 20 years old, with no major differences between males and females.

## Appendices

Data collection instrument

Date: Patient No.: Record:

General Data:

First Name(s): Last Name(s):

Sex: Male ( ) Female ( )

Age (years):

Severity Scale Score:

Diseases associated with healing defects:

Diabetes ( ) Congestive heart failure ( ) Hypertension ( ) Liver

diseases ( ) Ehlers-Danlos syndrome ( ) Keloid ( )

Autoimmune disease ( )

Bacteriological culture result:

*S. aureus* ( ) *S. pyogenes* ( ) Both ( ) None ( )

Adverse effects of the medication:

Rash ( ) Headache ( ) Nausea ( ) Abdominal pain ( ) Burning sensation

( ) Cellulitis ( ) Dizziness ( ) Ulcerative lesion ( ) Dry skin ( ) Others:

Product application instructions

I. Keep the cream at room temperature.

II. At the time of the visit, it is important to bring the medication to determine if it is being used.

III. Use neutral soap to wash the wound before applying the cream.

IV . Apply 2% mupirocin cream in the morning, afternoon, and evening for 10 days. Avoid contact with the eyes.

V . Do not bathe, wash, or swim for at least two hours after applying the medication.

VI. Do not apply any other antibacterial medication or any other product (gel, creams, lotions, ointments, etc.) to the affected areas while participating in the study.

VII. Inform the doctor if irritation, severe itching, rash, or no improvement occurs between the third and fifth day.

VIII. It is important to report the appearance of fever, general discomfort, or other symptoms.

Patient data collection instrument

Start date of treatment:

Follow-up visit date:

End date of treatment:

Control visit date:

Follow-up Days

Day 1

●Date: _____

●Photo:_____

●SIRS Measurement:_____

●Sample Collection:_____

●Medication Delivery: _____

Day 2

●Call:_____

●Medication Applied: _____

Day 3

●Medication Applied: _____

Day 4

●Medication Applied: _____

Day 5

●Visit:_____

●Photo:_____

●SIRS Measurement:_____

●Culture Result:_____

Day 6

●Medication Applied: _____

Day 7

●Medication Applied: _____

Day 8

●Call:_____

●Medication Applied: _____

Day 9

●Medication Applied: _____

Day 10

●Last Application: _____

●Medication Withdrawal:_____

Day 11

●Photo:_____

●SIRS Measurement:_____

Skin Infection Severity Scale

**Table 3:** Skin Infection Severity Scale (SIRS).

Signs and symptoms	Score	Definition
Blister	0 = absent	No evidence of blister
	1 = mild	Few vesicles present
	2 = moderate	Abundant vesicles
	3 = severe	Large areas covered with vesicles that may include large blisters
Exudate/Pus	0 = absent	No evidence of exudate or pus
	1 = mild	Small amounts of pus or secretions
	2 = moderate	The exudate or pus from the infected area is moderate
Scab	0 = absent	No evidence of scabs
	1 = mild	Evidence of some scabby areas
	2 = moderate	Scabs are present in the infected area
	3 = severe	Think scab appears in the impetiginized area
Erythema/Inflammation	0 = absent	Normal skin tone or color; no signs of erythema or inflammation
	1 = mild	Pinkish skin, with minimal signs of inflammation
	2 = moderate	Red skin, with minimal signs of inflammation
	3 = severe	Red skin with severe signs of inflammation
Scratching/Pain	0 = absent	No signs of scratching or indication of pain
	1 = mild	Some evidence of scratching, and the patient reports mild discomfort
	2 = moderate	Evidence of scratching, and the patient has painful lesions
	3 = severe	Evidence of excessive scratching, and the patient reports that the pain interferes with physical activities and sleep
